# Supremacy of nanoparticles in the therapy of chronic myelogenous leukemia

**DOI:** 10.5599/admet.2013

**Published:** 2023-09-26

**Authors:** Gopalarethinam Janani, Agnishwar Girigoswami, Koyeli Girigoswami

**Affiliations:** Medical Bionanotechnology, Faculty of Allied Health Sciences, Chettinad Hospital and Research Institute, Chettinad Academy of Research and Education, Chettinad Health City, Kelambakkam, Chennai-603103, India

**Keywords:** philadelphia chromosome, bionanotechnology, tyrosine kinase pathway, half-life, passive targeting

## Abstract

**Background and purpose:**

The reciprocal translocation of the ABL gene from chromosome 9 to chromosome 22 near the BCR gene gives rise to chronic myelogenous leukemia (CML). The translocation results in forming the Philadelphia chromosome (BCR-ABL) tyrosine kinase. CML results in an increase in the number of white blood cells and alteration in tyrosine kinase expression. CML prognosis includes three stages, namely chronic, accelerated, and blast. The diagnosis method involves a CT scan, biopsy, and complete blood count. However, due to certain disadvantages, early diagnosis of CML is not possible by traditional methods. Nanotechnology offers many advantages in diagnosing and treating cancer.

**Experimental approach:**

We searched PubMed, Scopus and Google Scholar using the keywords Philadelphia chromosome, bionanotechnology, tyrosine kinase pathway, half-life, passive targeting, and organic and inorganic nanoparticles. The relevant papers and the classical papers in this field were selected to write about in this review.

**Key results:**

The sensitivity and specificity of an assay can be improved by nanoparticles. Utilizing this property, peptides, antibodies, aptamers, etc., in the form of nanoparticles, can be used to detect cancer at a much earlier stage. The half-life of the drug is also increased by nanoformulation. The nanoparticle-coated drugs can easily escape from the immune system.

**Conclusion:**

Depending on their type, nanoparticles can be categorized into organic, inorganic and hybrid. Each type has its advantages. Organic nanoparticles have good biocompatibility, inorganic nanoparticles increase the half-life of the drugs. In this review, we highlight the nanoparticles involved in treating CML.

## Introduction

Hematopoietic stem cells (HSCs) are self-regenerating cells and can develop into different hematopoietic cells throughout a solitary life cycle and the process is termed hematopoiesis. Blood tumors can have various causes, one of which is hematopoiesis dysregulation. At various stages of hematopoietic development, mutations or other adverse occurrences may occur, leading to various tumor subtypes with various clinical signs. Hematological cancers can largely be divided into two categories, namely myeloid and lymphoid, depending on the origin of stem cells. Polycythemia, acute myeloid leukemia (AML), idiopathic thrombocytosis, chronic myeloid leukemia (CML), primary myelofibrosis, and myelodysplastic syndrome are examples of myeloid tumors that largely grow in the bone marrow [1]. With acute lymphocytic or lymphoblastic leukemia [ALL] being the most prevalent leukemia in juvenile, acute leukemia advance quickly in the creation of these immature blood cells. As opposed to acute leukemia, chronic leukemia advances more gradually and mainly affects adults. Chronic myeloid leukemia (CML) affects stem cells that differentiate into myeloid cells, which produce platelets, red blood cells (RBCs), and some types of white blood cells [2]. CML results from overactive tyrosine kinase that phosphorylates proteins and inhibits apoptosis and rapid cell growth [3]. In CML, reciprocal translocation occurs involving the fusion of the Abelson oncogene (ABL) on chromosome 9q34 with the breakage cluster area (BCR) on chromosome 22q11.2 (the Philadelphia (Ph) chromosome) [4]. Tyrosine residues found on the structure of ABL kinase serve as substrates for phosphorylation reactions that maintain the enzyme’s functional conformity. BCR coiled-coil domains encourage intermolecular cross-phosphorylation upon ABL kinase in the fused conformity, increasing its kinase activity and encouraging a continuous active tyrosine kinase [3,5]. The antiapoptotic sequels result from the p38 and JNK pathway suppression, p53 and Apaf-1 mutations, and an insufficiency in FASR. The proteins that promote cellular proliferation activate the RAS, MEK, Erk, Stat5, and PI3K/AKT signaling pathways [3].

In CML, the percentage of white blood cells increases. Depending on the characteristics, chronic myeloid leukemia can be divided into three stages: chronic, accelerated, and blast [4,6]. The majority of CML patients initially show up in the benign chronic phase (CP). If ignored, the disease will advance toward the myeloid or lymphoid blast phase (BP) where acute leukemia is reflected [4]. Diagnosis of CML includes complete blood count, CT scan, and biopsy. However, due to certain limits, early cancer detection is not possible by traditional methods. The sensitivity and specificity of an assay can be achieved by nanoparticles because of their large surface area. Utilizing this property, the nanoparticles are covered with peptides, antibodies, aptamers, etc., enabling cancer detection at a much earlier stage [7,8]. Nanoparticles are also employed in various fields of biomedicine, such as biosensing [9-12], targeted drug delivery [13-15], theranostics [16,17], photodynamic therapy [18], nanoformulation of nutraceuticals [19-21], tissue engineering [22,23], multimodal imaging [24,25], and also treating cancer [26]. They specifically target cancer because of their size, optical and catalytic properties [27,28]. The nanoparticles which are employed in treating CML are majorly discussed here.

## Clinical features, conventional and nano-enabled CML diagnosis

Fifty percent of CML patients do not show symptoms and are discovered by chance during standard checkups. Even when they have symptoms, they are not always identifiable [6]. The symptoms are early satiety, pain in the left upper quadrant, splenomegaly, dyspnoea, strain, or weariness caused by anemia [29]. In CML, there is surplus production of white blood cells due to the unrestricted multiplication of mature granulocytes and their precursors. It is a bi or tri-phasic disease and 20 % of patients usually die in the chronic phase. In the chronic phase of CML, the proliferation is under the control of the growth factor. In 80 % of cases, it proceeds to the accelerated and blast transformation phase [1]. Multidrug-resistant CML is encoded by the MDR1 gene, which works to actively transport medications out of cells to reduce intracellular drug concentration. This upregulation of MDR1 worsens treatment resistance, which raises the risk of cancer recurrence, indicating a poorer prognosis [30]. Atypical chronic myeloid leukaemia (aCML) is an uncommon form of myelodysplastic syndrome (MDS)/myeloproliferative neoplasm (MPN) that is BCR-ABL1 negative. It is historically known for having a poor prognosis. The difficulties in managing aCML affect both diagnosis and treatment options because there are no current guidelines for care and the disease’s clinical characteristics are heterogeneous [1].

Mostly CML is diagnosed from the complete blood count. Other diagnostic methods include fluorescent in situ hybridization, CT scan, biopsy, bone marrow aspiration, and quantitative RT-PCR. Atypical CML is diagnosed by neutrophilic leukocytosis and pronounced dysgranulopoiesis. Additionally, the WBC should be more than 13×10^9^ L^-1^ with more than 10 percent immature granulocytes and 20 percent blasts in the bone marrow and blood to meet the diagnostic criteria [1]. Diagnostic nanoparticles are intended to highlight irregularities and advance understanding of the fundamental physiological principles that underlie various diseases and treatment options [3]. In CML diagnosis, various inorganic nanoparticles are employed. A localized surface plasmon resonance layer made of polydimethylsiloxane (PDMS) supporting layer and gold nanoparticles (Au NPs) coupled to tumor necrosis factor (TNF) for cytokine detection is a microfluidic layer that traps and incubates cells. When compared to ELISA, PDMS uses a 100 times smaller volume of sample and the detection time also was 3X faster. Therefore, this novel optofluidic method was first used to detect THP-1 cells (human myeloid leukemia mononuclear cells) [31]. Size-dependent fluorescence Raman scattering was noticeably improved in surface-enhanced Raman scattering (SERS). Ag NPs-EIII1 network was created as an SERS probe using an Ag NPs assembled phage clone to recognize the cancerous cells [32]. The use of fluorescent Platinum nanoclusters (Pt NCs) instead of additional fluorescent biomarkers has shown considerable promise in the diagnosis of hematological disorders such as leukemia, lymphoma, and myeloma. K562 leukemia cell lines were co-cultured with bifunctional polyethyleneimine (PEI)-encased Pt NCs to show that Pt NCs smaller than 2 nm could specifically identify K562 cells with the internalized proportion at 97±4 % as opposed to peripheral blood mononucleated cells (PBMCs) at 20±8 % [33]. The BioCodeAunanobeacon, made up of AuNPs functionalized with hairpin-shaped string DNAs with a fluorophore on the extremes, is another sensor for identifying BCR-ABL. AuNPs may operate as a dark quencher on the single-strand fluorophores because of their LSPR. When no target was present, the hairpin maintained its tight conformation, keeping the fluorophore close to the AuNP, quenching the fluorescence. Instead, when there was a binding between the hairpin and its target, the hairpin opened, and the separation from the AuNP enabled the fluorescence to be detected [34].

## Upper hand of nanotechnology in CML treatment

**Figure 1.**
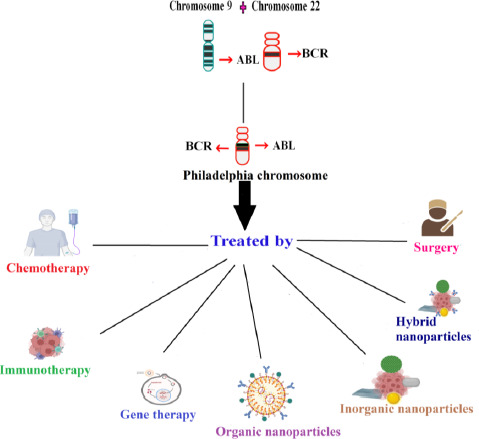
The various treatments involved in treating chronic myelogenous leukemia

Traditional CML treatment involves chemotherapy, radiotherapy, phototherapy, immunotherapy, etc. Chemotherapy drugs like cisplatin and carboplatin have side effects because of their neurology and nephrology-related toxicity [35]. In targeted therapy, ligands were combined with chemotherapy drugs to treat the disease. In photothermal therapy, electromagnetic radiation was used. They excite the non-toxic material known as photosensitizer to generate ROS, which targets and kills the cancer cells [36]. Despite the availability of different types of treatment for CML, nanomedicine has an added advantage because of the size of nanoparticles and the ability to target cancer cells alone [37]. Nanotechnology successfully ameliorates drug uptake issues and helps maintain the drug release by increasing its half-life. The half-life is usually arbitrated by the macrophage. They carefully acknowledge the cancer cells and deliver the drugs to the target location accurately, minimising the faulty site of action in the cells. Thus, nanotechnology helps battle the shortcomings of conventional treatment procedures [38]. Nanoparticles are good in treating cancer due to their distinctive characteristics. Different types of nanoparticles have their advantage. Organic nanoparticles have excessive biocompatibility in delivering drugs. Inorganic nanoparticles increase the half-life more when compared to other types of nanoparticles [36]. Nanoparticles coated with hydrophilic polymer curtail opsonisation and escape from the immune system. Passive targeting is the mechanism by which drugs are delivered through blood vessels. Thus, negatively charged liposomes ranging between 50 to 100 nm in size increase the drug uptake by flowing freely and by having high surface energy [3]. Thus, nanomedicine serves as one of the effective treatments in treating cancer. Figure 1 depicts the various treatments for treating chronic myelogenous leukemia.

## Inorganic nanoparticles in CML therapy

Only six different types of inorganic nanoparticles—zinc oxide, copper, gold, silver, ferrous oxide, and carbon nanotubes (CNTs)—have been investigated as potential ways to deliver drugs to CML. Silver nanoparticles bring about cytotoxicity effect in CML cells [6]. Gold nanoparticles (GNPs) have been proven to be ideal drug nanocarriers for anticancer medications because they can safely transport and protect a large therapeutic payload and deliver the medication just to the tumor site using active/passive targeting techniques, increasing the therapeutic index [39]. A class of magnetic nanoparticles (Magnetic NPs) that exhibit small size, quantum size, increased surface area, and macroscopic quantum tunneling phenomena are predominantly made of Fe oxide at the nanoscale. The inherent biological functional components make them suitable for disease monitoring and diagnosis [40,41]. Leukemia cells’ proliferation was inhibited by carbon-nanostructured C60 fullerene, which showed strong anticancer properties. It got activated by violet light to produce a lot of ROS and apoptosis [42]. Over the past ten years, there has been a lot of curiosity about the prospective uses of hybrid nanocomposites. Due to the additive and mixing qualities of inorganic and organic components, these composites have intriguing features. Various nanoformulations of inorganic nanoparticles in treating CML are discussed here.

Exosomes released by CML cells have been shown in numerous publications to be able to stimulate the growth of new blood vessels, pointing to a potential function in neo-angiogenesis [43]. Exosomes purified from K562 cells encouraged the expansion of newly created vasculature, accompanied by an uptick in VEGFR1 expression. In a chorioallantoic membrane (CAM) model, it has been shown that gold nanoparticles (AuNPs) enriched with antiangiogenic peptides can prevent angiogenesis by having an impact on the intrinsic VEGFR pathway [44]. In CML K562 cells, CuO-TiO_2_-Chitosan-Berbamine nanocomposites led to the death of cells in a dose-dependent way with an IC50 of 113.54 g/mL. By targeting members of the BCL-2 family (BAX and BCL-2) and raising the level of p53, they cause mitochondrial apoptosis [45]. Dasatinib-loaded gold nanoparticles (DSB-GNPs) are a reliable method for DSB administration in the therapy of CML due to their claimed cytotoxic effect, systemic bioavailability, and sustained release [46]. After treating K562 cells with zinc oxide NPs (ZnO NPs), researchers could detect changes in the transcriptome using a DNA microarray-based transcriptomic method. The transcriptomics results supported the ZnO NPs’ induction of apoptosis in K562 cells and suggested potential molecular pathways by which the NPs caused toxicity in the leukemic cells [47]. Various inorganic nanoparticles involved in nanoformulation for treating CML have been summarized in Table 1.

## Nanoparticles targeting tyrosine kinase pathway

Tyrosine kinases (EGFR, FGFR, PDGFR, and VEGFR, for example) are commonly abnormally triggered in tumors and numerous publications have seen encouraging therapeutic benefits by employing very small-molecule that block the tyrosine kinase key pathways associated with the growth and progression of tumor cells [58]. An actively functioning non-receptor tyrosine kinase enzyme called Bcr-Abl can be identified in the cytoplasm of leukemia cells that have the Philadelphia chromosome [59]. Tyrosine kinase inhibitor (TKI) based therapy is regarded as the most effective form of management for CML. In CML, the RAS mitogen-activated protein kinase family (MAPKs), including the ERK1/2, MEK, JNK, and p38 families, is a key signaling cascade that undergoes modifications [60]. Tyrosine locations in BCR-ABL1’s active conformation, like Y412 in the activation loop and Y245 in the SH2-kinase linker, are known to control the activity and function of tyrosine kinase. The Y177 binding site on the BCR domain is recognized by the SH2-domain protein growth factor receptor bound protein-2 (GRB2), which supports RAS activation through Son of Sevenless (SOS), a guanine nucleotide exchange factor protein and the scaffolding adapter GAB2 (GRB2-associated binding protein 2). These three factors work in concert to facilitate CML [61]. Two isoforms of AKT, a serine/threonine kinase from the AGC family of kinases, are expelled in hematopoietic stem cells. P-AKT, which has been activated, followed by PI3K activation by BCR-ABL1, can prevent apoptosis and promote cell growth [62]. By examining PTEN’s role in BCR-ABL showing Ba/F3 cells, it has been discovered that it causes the reduced activity of PTEN phosphatase and a decrease in the production of the p53 protein [63].

**Figure 2.**
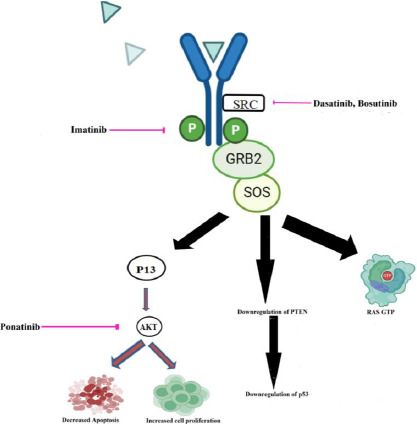
Schematic representation of the nanoformulated chronic myelogenous leukemia drugs inhibiting BCR-ABL tyrosine kinase

Various nanoformulations of drugs that target the tyrosine kinase pathway, which plays a role in CML, have been discovered. The Bcr-Abl tyrosine kinase protein is the principal biological target of ponatinib (Pon). It targets the Akt pathway when encapsulated into reproducible biomimetic lipid nanoparticles [64]. Gold nanoparticle-encapsulated dasatinib competitively inhibits the ATP site of SRC. High-density lipoprotein nano-formulated bosutinib inhibits Bcr-Abl and SRC kinase [65]. Imatinib encapsulated by polymeric nanoparticles inhibits the phosphate group transfer to tyrosine and subsequently inhibits its activation [66]. Figure 2. schematically represents the nanoformulated chronic myelogenous leukemia drugs inhibiting BCR-ABL tyrosine kinase.

## Organic nanoparticles in CML therapy

Organic nanoparticles include lipid-based, polymer-based, dendrimers, etc. The superiority of liposomes over free pharmaceuticals in drug delivery can be due to their lower toxicity, the potential to couple them with antibodies, additional signalling molecules for more focused distribution, and enhanced altered pharmacokinetics and bioavailability [67]. Therapeutic medicines can be incorporated into polylactic-co-glycolic acid (PLGA), which has various benefits, including long-term release and enzymatic degradation resistance, and can be used to deliver pharmaceuticals effectively and with less toxicity to normal cells [68]. Polymer micelles are created using the water-soluble building block polyethylene glycol (PEG). PEG has the benefits of making micelles biocompatible and increasing the bioavailability of some medications [69]. Small NPs known as dendrimers, exhibit radial symmetry and function as an effective drug delivery vehicle [70, 71]. Due to their advantages, organic nanoparticles are employed in treating various diseases. The nanoparticles which are employed in treating CML are discussed here.

To target the overexpression of somatostatin receptor type 2 in CML, amino-PEGylated quantum dots nanoparticle was succinylated and coated with the ligand octreotide [72]. K562 cells treated with dendrosomal nano-formulated solanine (DNS), DNS+ imatinib showed increased apoptosis and inhibited the expression of c-MYC, hTERT, NF-kB, mTOR, PI3KCA and also dephosphorylated the AKT protein [73]. Multidrug resistance (MDR) in CML was overcome by co-encapsulation of mitoxantrone and a P-glycoprotein(P-gp) inhibitor in β-elemene, the solid lipid nanoparticles. The MDR was overcome by blocking intracellular ATP production and P-gp release by β-elemene [74]. CML stem cells take up the poly(lactide co-glycolic acid) (PLGA) nanoparticle construct containing polymethine dye DY-635 through the anion transport protein 1B3 (OATP1B3). This transporter was activated via hypoxia-inducible factor 1α. Thus, the CML stem cells could be targeted via this drug-loaded nanoparticle containing DY-635 dye [75]. When given a high dose of amphiphilic polymer which was conjugated and self-assembled into nanoparticles, its half-life increased by approximately 20 fold and it was effective in inhibiting Bcr-Abl [76]. When K562 cells were administered with 160 μM of hydroxylated fullerene, it showed a decrease in the mitochondrial membrane potential, a change in morphology, and the G2-phase arrest [77]. CML stem cells aberrantly express CD26. Liposomes conjugated with Begelomab (anti-CD26) loaded with venetoclax at 100 nM concentration significantly reduced the expression of CD26 in both stem cells and progenitor cells [78]. Anti-Bcr-Abl antibodies loaded in poly(d,l-lactide-co-glycolide) nanoparticles (PLGA NPs) effectively degraded the Bcr-Abl oncoprotein in CML cells [79]. Various organic nanoparticles involved in treating CML have been summarized in Table 2.

## Conclusion

Recent advances in nanotechnology have sparked the creation of numerous improvised nanoparticles for diagnostic and therapeutic uses. Of all newly reported cancer cases in India, 8% are blood cancer cases. Blood cancers like leukemia, lymphoma, and myeloma are more prevalent in men than women. Several improvements in treating CML patients have improved. An effective tool for treating is provided by nanotechnology. Nanomedicines can target and regulate the release of drugs in diseased areas and protect the body from deadly off-target adverse effects. Nanomedicines employed in CML target tyrosine kinase pathway, apoptosis, and multidrug-resistant gene MDR1. Due to their large surface area, they are coated on the biosensors to detect the malignant cells precisely with high sensitivity and specificity, enabling early-stage cancer detection.

## Figures and Tables

**Table 1.** Explanations of the various nanoformulations of inorganic nanoparticles that have been employed in CML treatment so far

No.	Nanoparticle type	Conjugated to	Mechanism of action	Experiment	Ref.
1	Mesoporous silica nanoparticle	Glycine and doxorubicin	59 % of doxorubicin drug is released from the aggregate inhibiting the K562 cells.	*In vitro*	[48]
2	Thioxanthone derived gold nanoparticle	Doxorubicin	5μl of Conjugated doxorubicin killedK562 cells with 7.3 % cytotoxicity.	*In vitro*	[49]
3	Gold nanoparticle	AS1411, Doxorubicin, Anti-221	They triggered apoptosis of drug-resistant CML cells by down-regulating DNMT1 and miR-221.This downregulation restored p15ink4b andp27kip1 tumor suppressor expression. They also restored P-gp expression which was downregulated by miR-221.	*In vitro*	[50]
4	Cobalt ferrite nanoparticle	45 % oleic acid	9μM of coated CoFe_2_O_4 s_howed strong apoptotic effects on K562 cells when they aretreatedfor72 h.	*In vitro*	[51]
5	Arsenic trioxide nanoparticle	DSS6 and F56	20 percent F56 and 50 percent DSS6 oligopeptide target the vascular and endothelia niches. They suppress the colony-forming function and proliferation of K562 cells with the IC50 value of 3.29 μM.	*In vitro*	[52]
6	Realgar	Imatinib	They suppressed the tyrosine kinase activity via apoptotic mechanism.	*In vitro*	[53]
7	Arsenic sulfide nanoparticle	Water dissolved	They directly degraded the BCR-ABL by down-regulating hypoxia-inducible factor 1α and ROS.	*In vitro*	[54]
8	Ferrous oxide and zinc oxide nanoparticle	Hydroxylated carbon nanotube	The proliferation activity of K562 cells is decreased through activation of apoptosis and G1 arrest. It is carried out by upregulating the FOXO3a and SIRT1.They also inhibit the NF-Kβ signaling pathway.	*In vitro*	[55]
9	Silver nanoparticle	*Sophora pachycarpa* extract	It showed anticancer activity in K562 cells with an *IC*_50_ value of 19.5 mg/ml.	*In vitro*	[56]
10.	Ultra small platinum nanoparticles	Gold nanorods	It facilitated ROS fluctuation and triggered cellular autophagy by increasing the level of Beclin-1, which degraded the BCR-ABL. It also downregulated the phosphorylation of PI3K and AKT.	*In vitro*	[57]

**Table 2.** Summarises the other organic nanoparticles involved in CML treatment

No.	Nanoparticle type	Conjugated to	Experiment	Mechanism of action	Ref.
1	Lipid nanoparticle	siRNA	*In vivo*	The Bcr-Abl fusion oncogenein mousemyeloid leukaemia model is targeted by lipidloaded nanoparticle containing Bcr-Abl siRNA.	[80]
2	Co-polymer, poly oligo(ethylene glycol) methacrylate-b-poly(styrene–co-4-formylphenyl methacrylate) with bottlebrush-like oligo (ethylene glycol) methacrylate	CHMFL-ABL-053	*In vivo* and *in vitro*	When this micelle is given in high doses it enables controlled release and longer circulation of drugs and makes it a potential CML drug candidate.	[81]
3	Protein scaffold	C- terminus of Bcr-Abl is attached to Arg-repeating hexapeptide (R6)	*In vivo* and *in vitro*	A powerful dodecameric peptide antagonist of p53-MDM2/MDMX interaction (PMI), found at the N-terminus of Bcr/Abl, with its Arg-repeating hexapeptide inhibits the MDM2/MDMX and activates the p53 pathway.	[82]
4	Ferritin dendrimer	pre-miRNA	*In vitro*	The leukaemia cells internalize the pre-miRNA by expressing CD71 receptor. Once internalised the pre-miRNA matures and brings about morphological changes.	[83]
5	Polyethylenimine-Cholestrol	siRNA	*In vitro*	The PEI-Chol mediated distribution of Bcr-Abl siRNA resulted in increased level of apoptosis of K562 cells.	[84]
6	Lipid-polymer hybrid	Doxorubicin and gallic acid	*In vivo* and *in vitro*	They reduced the size of tumor growth in K562 cells from 956 mm^3^ to 213 mm^3^.	[85]
7	Polylactic acid nanoparticle	Daunorubicin and glycyrrhizic acid	*In vitro*	The drug caused apoptosis of K562 cells.	[86]
